# The Question of Alcohol

**Published:** 1919-07-19

**Authors:** 


### The Hospital

The Workers' Newspaper of Administrative Medicine and Institutional Life, Administration, National Insurance and Health.

No. 1728, Vol. LXVI. SATURDAY, JULY 19, 1919. PRICE TWOPENCE

### THE QUESTION OF ALCOHOL.

There are indications that the old controversy between the "temperance" fanatics, or "prohibitionists" as they are now called, and their opponents is about to be aired once more in the daily press, and we are told that our own temperance organisations are to receive financial support from the American continent. Those who are inclined to make light of this American interference are likely to be surprised when the promised campaign really starts, for the fanatic invariably makes converts, particularly when he can afford to buy them.

In a report of the conference of the British Temperance League held at Bradford, we observe that the speakers, as is their wont, displayed extraordinary ignorance of the first principles of logic. Professor Sir G. Sims Woodhead attempted to show the advantage of a "dry" Army; if he had seen the effect of enforced "dryness" on the American Army and Navy he might, perhaps, be less ready to dogmatise now. The great mistake that was made by our Army authorities—or was it the Government?—during the war was that they did not provide a daily issue of beer and stout for the men in and near the line. Most of the officers' messes could obtain whisky even when actually in the line, but the men had to content themselves with tea, which, in its Army form, is positively injurious, whereas beer and stout are not. Practically all medical officers, who have had any experience of front-line work, agree that, had our troops been liberally supplied with good beer and stout, not only would they have been more contented, but their resistance to staphylococcal infections of the skin, to which lousiness, scabies, and lack of washing facilities rendered them liable, would have been increased. Professor Woodhead, in referring to the rum ration, makes the obvious remark about "Dutch courage"; as a matter of fact, when rum was issued at all, at any rate during the latter part of the war, it was given to the men during their brief spells of rest, and no reasonable person could grudge it them.

Sir Victor Horsley, to whose unselfish patriotism and untiring energy in the Eastern theatre of war we can personally testify, died because, being a teetotaller, he thought he was proof against heat-stroke, and we may here take the opportunity of pointing out that, in the opinion of most people who have lived in the East, a moderate quantity of alcohol, preferably in the form of whisky, is almost essential for Europeans. Certain is it that European women, most of whom would never take spirits in their own country, find that, by taking a little whisky with their meals and at night-time, they can avoid the unpleasant digestive disturbances to which they are so liable in the tropics. Medical men know better than most people the dangers of alcohol, but most of them will surely agree with us that the remedy for alcoholism lies not in enforced prohibition. To describe beer as "a poisonous drug" is, on the face of it, ridiculous. Let the sale of cheap, inferior spirits be forbidden for all time, but forbid also the reformer's claptrap about the dangers of beer-drinking. In fact our temperance leagues would be better employed in lecturing the people on the dangers of over-eating, or the necessity of keeping their teeth clean and their bowels open, and on the importance of fresh air and outdoor games.

